# Impact of the Coronavirus Disease 2019 (COVID-19) Pandemic on Cervical Cancer Screening in Gynecological Practices in Germany

**DOI:** 10.3390/cancers14194820

**Published:** 2022-10-02

**Authors:** Niklas Gremke, Sebastian Griewing, Markus Felgentreff, Karel Kostev, Matthias Kalder

**Affiliations:** 1 Department of Gynecology and Obstetrics, University Hospital Marburg, Philipps-University Marburg, Baldingerstraße, 35043 Marburg, Germany; 2 Epidemiology, IQVIA, Main Airport Center, Unterschweinstiege 2-14, 60549 Frankfurt am Main, Germany

**Keywords:** COVID-19 pandemic, cervical cancer, screening, gynecological practices, Germany

## Abstract

**Simple Summary:**

The outbreak of the coronavirus disease 2019 (COVID-19) is a major challenge for healthcare systems and impedes cancer screening programs worldwide. However, no research has been performed that analyzes the effects of the COVID-19 pandemic on the newly implemented German Cervical Cancer Screening (CCS) program. Here, we showed a severe clinically relevant decrease of patients receiving CCS in Germany between the pre-pandemic time period of 2018–2019 and the pandemic years of 2020–2021. Given that this sudden drop in number of CCS per practice is unlikely, our data suggest a dramatic number of underdiagnosed cervical precancer lesions that may lead to increased cervical cancer burden in the future.

**Abstract:**

**Purpose**: the aim of this cross-sectional study was to analyze the impact of the COVID-19 pandemic on Cervical Cancer Screening (CCS) in gynecological practices in Germany. **Methods**: The basis of the analysis was the Uniform Evaluation Standard (EBM) of the Statutory Health Insurance Scheme. This cross-sectional study included all women aged ≥20 years with at least one CCS (clinical and cytological examination) in 223 gynecological practices in Germany during the period 2018–2021. The number of patients with CCS per practice was shown for each year. The average number of patients per year was compared between the pre-pandemic time period (2018, 2019) and the pandemic time period (2020, 2021) using Wilcoxon tests. Analyses were conducted separately for clinical investigations and cytological investigations and were also stratified by age group (20–34, 35–50, >50 years). **Results**: CCS in gynecological practices significantly decreased in Germany between the pre-pandemic time period of 2018–2019 and the pandemic years of 2020–2021. This decrease was observed in all age groups but was stronger in women aged 20–34 (−25.6%) and weaker in women aged >50 (−15.2%). **Conclusions**: We found a statistically and clinically relevant decrease of patients receiving CCS in gynecological practices in Germany. This finding is even more exceptional because the new screening algorithm with direct invitations for each patient started in 2020 and was supposed to lead to a higher number of patients in its first years. However, the observed decline in the detection of cervical precancer lesions may lead to increased cervical cancer burden. Risk-based screening strategies and further measures are necessary to adapt to the ongoing COVID-19 pandemic and return to pre-pandemic CCS numbers.

## 1. Introduction

Cervical cancer (CC) is the fourth most common cancer diagnosed in women worldwide, with approximately 604,000 new cases and 342,000 deaths in 2020, and affects primarily middle-aged women in low- and middle-income countries [1,2]. In Germany, the incidence and mortality rates of CC decreased dramatically following the establishment of a cervical cancer screening (CCS) program in 1971, which included annual cytological screenings (Pap smear) and clinical examinations (vaginal examination using a speculum, palpation of lymph nodes, etc.) as an offer from the Statutory Health Insurance Companies [3,4]. However, the incidence rate for CC (9.3 per 100,000 women), the mortality rate (2.6 per 100,000 women), and the CCS participation rate (between 44.3 and 56.9% in 2003) have nearly stagnated in recent years [5,6]. Based on these issues, the Federal Joint Committee (G-BA) decided in 2016 to modify the opportunistic CCS program and implemented an organized population-based call–recall CCS program (beginning on 1 January 2020) with two new age-dependent screening algorithms to further improve the quality of CCS in Germany. While annual cytological screening and clinical examinations for women between 20 and 34 years would be continued, women older than 35 years would receive co-testing with cytology and an HPV test every three years [7,8,9]. Since the high-risk human papillomavirus (HPV) is known to cause cervical cancer in a multistage development initially confined to the cervical epithelium, early detection of precancerous cervical lesions (CIN) is critical for effective treatment and good prognosis [10,11,12]. Therefore, women who are never screened or who have an inadequate screening history have a higher risk for CC and are diagnosed in more advanced tumor stages with a worse prognosis [13,14].

In light of this issue, the COVID-19 pandemic has presented a major challenge for healthcare systems worldwide to ensure the care of COVID-19-infected patients while maintaining essential health services [15,16]. At the beginning of 2020, the German government decided to take drastic measures to reduce the number of COVID-19 infections. As a result, elective operations and non-urgent clinical visits were postponed, and a national wide lockdown was imposed to increase intensive care capacities [17]. The World Health Organization (WHO) conducted a rapid assessment survey of service delivery for noncommunicable diseases (NCDs) during the COVID-19 pandemic among 194 ministries of health. This survey discovered that postponements of national cancer screening programs (e.g., for breast and cervical cancer) occurred in more than 50% of all responding states, as well as growing evidence that disruptions of cancer screening programs would result in a strong increase of advanced cancer diagnoses and deaths in the future [18,19,20]. A recently published systematic review and meta-analysis of 39 publications analyzed the impact of COVID-19 on cancer screening worldwide and found an overall decrease of −51.8% (95% CI, −64.7% to −38.9%) for cervical cancer screening rates during the pandemic period compared with pre-pandemic levels [21].

However, to the best of our knowledge, no research has been performed that analyzes the effects of the COVID-19 pandemic on the newly implemented German CCS program. To bridge this gap, we aimed to analyze the number of women who received CCS per practice between 2020–2021 (pandemic period) and 2018–2019 (pre-pandemic period), using data from 223 gynecological practices in Germany in this cross-sectional study.

## 2. Materials and Methods

### 2.1. Database

This study used data from the Disease Analyzer database (IQVIA), which contains drug prescriptions, demographic data, and diagnoses anonymously obtained directly from computer systems used in general and specialized practices. The encoded data were transmitted to IQVIA every month. The DA database has been described in detail elsewhere [22]. The quality of the data is assessed regularly by IQVIA based on various criteria (e.g., completeness of documentation and linkage between diagnoses and prescriptions). Practices included in the Disease Analyzer database are selected based on several criteria, such as age of physician, specialty group, community size category, and German federal state. Previously, the panel of German practices included in the DA database has been shown to be representative for all general and specialized practices in Germany [22]. 

### 2.2. Study Population 

The basis of the analysis was the Uniform Evaluation Standard of the Statutory Health Insurance Scheme (Einheitlicher Bewertungsmaßstab, EBM). The EBM contains different services for cervical cancer screening that can be billed (fee schedule positions, GOP) by gynecologists: clinical examination (GOP: 01730 in 2018–2019 and 01760 and 01761 in 2020–2021) and cytological examination (GOP: 01731 in 2018–2019 and 01763 in 2020–2021).

This cross-sectional study included all women aged ≥20 years with at least one cervical cancer screening (clinical and cytological examination) in 223 gynecological practices in Germany in the years 2018, 2019, 2020, and 2021. Only practices continuously delivering data between January 2018 and December 2021 were included.

### 2.3. Statistical Analyses

The number of patients with cervical cancer screenings per practice was shown for each year. Finally, the average number of patients per year was compared between the pre-pandemic time period (2018, 2019) and the pandemic time period (2020, 2021) using Wilcoxon tests. Analyses were conducted separately for clinical investigations and cytological investigations and were also stratified by age group 20–34, 35–50, >50 years. *p*-values < 0.05 were considered statistically significant. Analyses were performed using SAS 9.4 (SAS Institute, Cary, CA, USA).

## 3. Results

### 3.1. Decreased Number of Women with Cervical Cancer Screening per Practice

**Figure 1:**
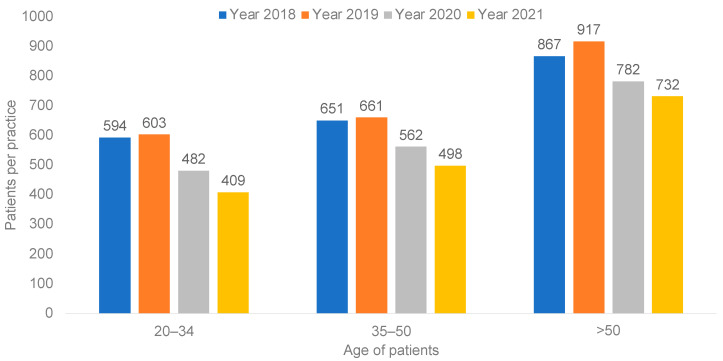
Number of women with cervical cancer screening per practice in Germany in the time period 2018–2021.

In total, 665,917 patients had at least one visit with one of the 223 gynecological practices in 2018, 672,640 in 2019, 658,732 in 2020, and 730,951 in 2021. The number of women per practice with one cervical cancer screening in 2018–2021 is shown in Figure 1. After a small increase in 2019, the numbers strongly decreased in 2020 and in 2021 from 603 in 2019 to 482 in 2020 and 409 in 2021 in the age group 20–34 years, from 651 in 2019 to 562 in 2020 and 498 in 2021 in the age group 35–50, and from 917 in 2019 to 782 in 2020 and 732 in 2021 in the age group >50.

### 3.2. Age-Stratified Differences in the Number of Women with Cervical Cancer Screening per Practice 

The overall difference in the average number of women newly diagnosed with cervical cancer screening per practice is displayed in Table 1. We observed a significant decrease in cervical cancer screenings between 2018–2019 and 2020–2021. This decrease was observed in all age groups but was stronger in women aged 20–34 (−25.6%) and weaker in women aged >50 (−15.2%). 

In terms of type of examination, the strongest decrease was found for cytological examination (−57.3%), where the decrease was stronger in women aged >50 (−61.8%) than in women aged 20–34 years (−47.5%). For clinical examination, the decrease was −17.8% and stronger in women aged 20–34 (−25.0%) than in women >50 years (−13,1%). All changes were statistically significant.

## 4. Discussion

In this study, we showed a strong decrease in women who received CCS per practice between 2020–2021 (pandemic period) and 2018–2019 (pre-pandemic period) for all age groups using data from 223 gynecological practices in Germany. In particular, the new German CCS algorithm recommends an annual Pap smear for women aged 20–35, while the co-testing interval was extended to 3 years for women aged older than 35 years. Of note is that the decrease in CCS per practice was much stronger in women aged 20–35 (−25.6%) compared with women aged >35 (−19.2%), pinpointing the profound negative impact of the COVID-19 pandemic on CCS rather than the consequences of the new screening algorithm. The significant decline in CCS rates is also in line with recent publications from the Unites States and Europe [23,24], with several reasons for this widespread negative effect of the COVID-19 pandemic on CCS programs being mentioned in the current literature. For example, stay-at-home orders from governments in response to waves of COVID-19 infections and hospitalizations, the postponement of non-urgent clinical visits to increase intensive care capacities, and patients’ fear of visiting medical facilities are major reasons for reduced screening services. The COVID-19 pandemic has also indirectly impeded screening capacities due to the lack of staff and pandemic-related supply shortages of consumables and reagents (e.g., for HPV PCR), leading to reduced laboratory testing volumes and capacities [10]. Notably, the suspension of cervical cancer screening programs hit those populations hardest, where cancer screening rates were the lowest to begin with [25,26]. This finding mainly applies to patients with low socioeconomic status and minorities, who thus have lower CCS participation rates and higher cancer incidence and rates of cancer-related deaths [26]. As a result, the ongoing COVID-19 pandemic has exacerbated these determinants on screening coverage, and vulnerable groups in our community are now faced with additional challenges to protect themselves against the coronavirus while also dealing with low income and impending unemployment [25]. In light of this issue, improving the resilience and equity of CCS in terms of the ongoing COVID-19 pandemic is an urgent goal. Recently, a plethora of opportunities, such as HPV-based screening with self-sampling, risk-based screening, and the use of telehealth, have been intensively discussed in the literature to improve access to CCS. The advantage of HPV testing alone is that it can be performed with samples collected by patients at home, thus reducing patient–provider contact, while large meta-analyses revealed that HPV–PCR from self-collected samples has the same accuracy as clinician-collected samples [27]. In particular, patients with negative HPV screens can safely delay CCS visits for some time. In the risk-based screening and management approach, patients are managed based on their screening results, with diagnosis and treatment being accelerated for high-risk patients and unnecessary diagnostic procedures being decreased for low-risk patients [28]. Both of the previously described measures can be supported via telehealth to manage self-collection of HPV samples. Finally, telehealth increases access to specialty consultation and, as a further benefit, screening results can be also discussed in virtual visits without the risk of COVID-19 exposure [29,30].

However, additional longitudinal research studies are needed to analyze the consequences of COVID-19-related suspension on the newly organized German CCS program with regard to screening factors such as women’s age and socioeconomic status, preferably using incidence rates for cervical precancer lesions and cervical cancer as primary outcomes.

### Strengths and Limitations

Our cross-sectional study has several strengths. The Disease Analyzer (DA) is a large German outpatient database that contains data from more than 200 gynecological practices. Furthermore, this study is the first large nationwide study to identify the severe impact of the COVID-19 pandemic on cervical cancer screening in outpatients treated in gynecological practices in Germany.

However, the study results should be interpreted in light of several limitations. The DA does not contain information on external confounding factors (e.g., alcohol, tobacco consumption, socioeconomic status), and no further patient information (e.g., HPV status, anamnestic data) is available. Second, changes in the German CCS program since 2020 were accompanied by new EBM numbers, therefore data for HPV tests and co-testing (cytology and HPV test) before 2020 were not available for this study. Third, results regarding Pap smears were lacking, and no indications for cytological and clinical examination were available. 

## 5. Conclusions

In our study, we observed a dramatic decrease in cervical cancer screenings for women of all ages who were treated in gynecological practices in Germany between the pre-pandemic time period of 2018–2019 and the pandemic years of 2020–2022. The severe impact of the COVID-19 pandemic on CCS may lead to increased cervical cancer burden in the future if CCS programs do not resolve backlogs with efficient and equitable recovery strategies. Finally, far-reaching measures are necessary to establish a more resilient CCS-program to anticipate possible disruptions in the future.

## Figures and Tables

**Table 1:** Age-stratified differences in the number of women with cervical cancer screening per practice in Germany between 2020–2021 and 2018–2019.

Examination	Age Group	2018, 2019 Mean Number of Women per Year	2020, 2021 Mean Number of Women per Year	Difference	*p*-Value
Cervical cancer screening	Total	2147 (1509)	1732 (1308)	−19.3%	<0.001
Cervical cancer screening	20–34	599 (468)	445 (389)	−25.6%	<0.001
Cervical cancer screening	35–50	656 (474)	530 (412)	−19.2%	<0.001
Cervical cancer screening	>50	892 (648)	757 (566)	−15.2%	<0.001
Clinical examination	Total	2051 (1263)	1687 (1204)	−17.8%	<0.001
Clinical examination	20–34	575 (416)	431 (353)	−25.0%	<0.001
Clinical examination	35–50	627 (411)	518 (390)	−17.5%	<0.001
Clinical examination	>50	849 (527)	738 (523)	−13.1%	<0.001
Cytological examination	Total	236 (1134)	101 (644)	−57.3%	0.003
Cytological examination	20–34	62 (298)	32 (213)	−47.5%	0.003
Cytological examination	35–50	70 (333)	28 (176)	−59.3%	0.001
Cytological examination	>50	104 (508)	40 (258)	−61.8%	0.010

Data are means (standard deviation); *p*-values were obtained using Wilcoxon tests.

## Data Availability

Anonymized raw data are available upon reasonable request.
